# Lumbar Herniation of Kidney following Iliac Crest Bone Harvest

**DOI:** 10.1155/2016/5365647

**Published:** 2016-11-30

**Authors:** Michael Justin Willcox

**Affiliations:** Tulane University School of Medicine, New Orleans, LA 70112, USA

## Abstract

The iliac crest is a popular source for autogenous bone harvesting, but the process is rife with complications. This case report presents a patient that experienced incisional lumbar herniation of her kidney following an iliac crest bone harvesting procedure. A discussion is included on the underappreciated complications of this procedure and recommendations for improving outcomes with more thorough evaluation and documentation.

## 1. Introduction

The iliac crest is the most popular source for autogenous bone for a wide variety of orthopedic grafting procedures, likely owing to its relatively easy access to an excellent supply of both cortical and cancellous bone. The harvesting process does however provide various potential complications that are likely underestimated in both frequency and severity [1, 2]. This case report presents an unusual complication following an iliac crest bone harvesting procedure and a discussion on the need to continue evaluating bone harvesting procedures for future improvement.

## 2. Case Presentation

**Figure 1:**
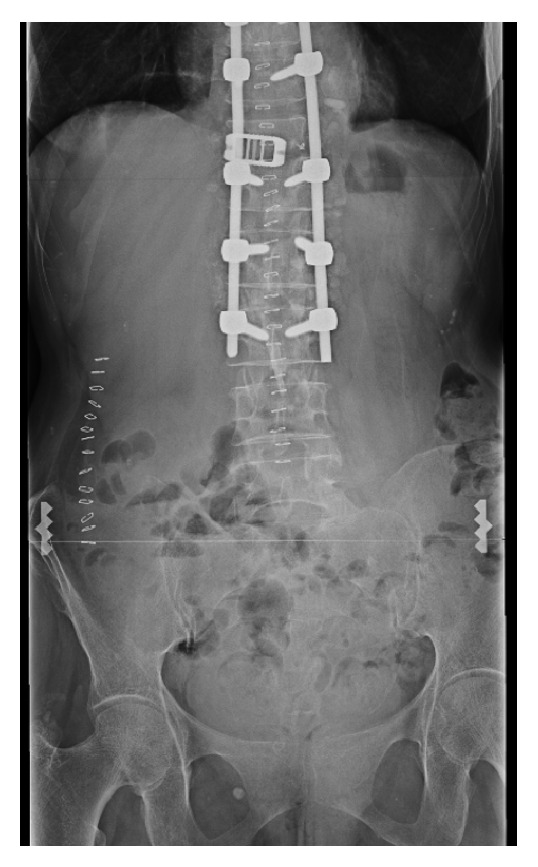
Initial iliac crest bone harvest procedure postoperative radiograph demonstrating relative position of incision with closure staples.

**Figure 2:**
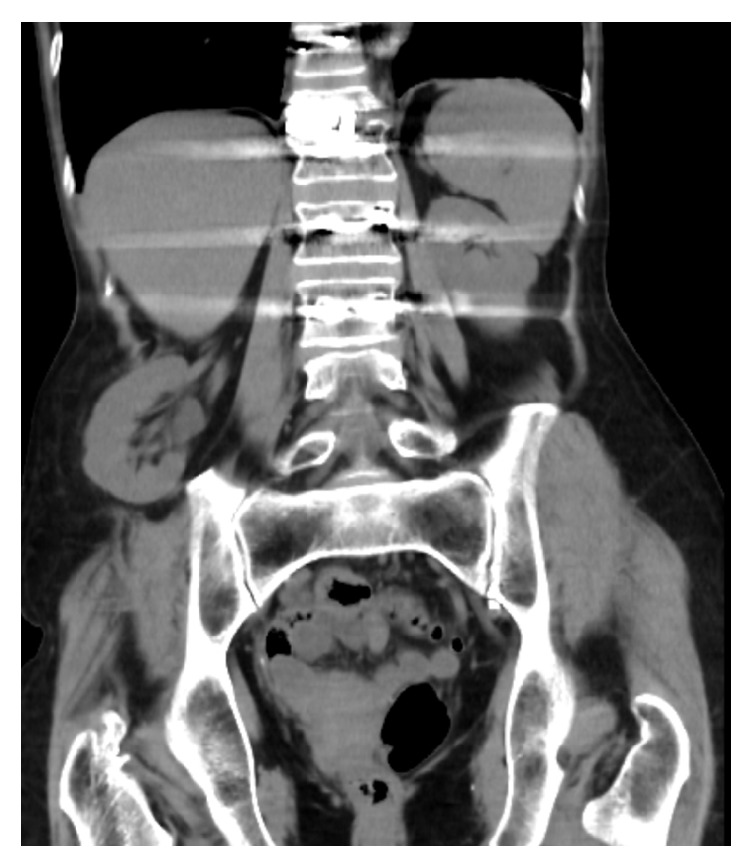
Coronal CT shows herniation of the right kidney immediately superior to the site of iliac crest bone harvest.

**Figure 3:**
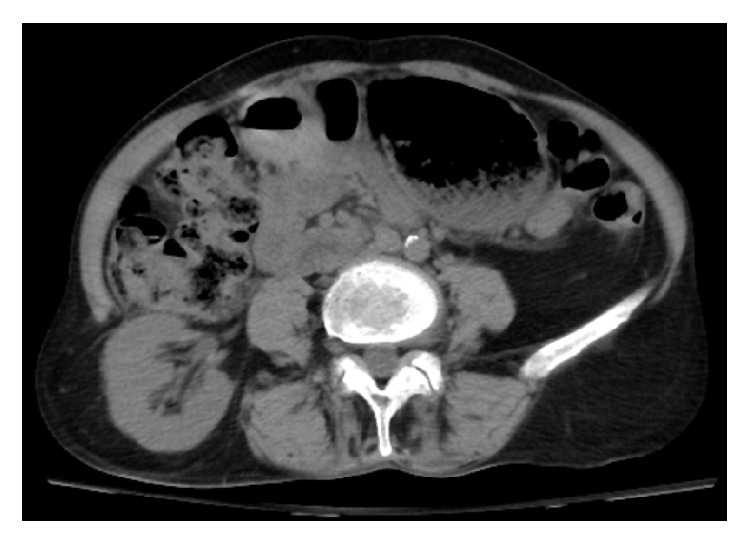
Axial CT demonstrates the extent of kidney herniation.

A 63-year-old Caucasian woman with a medical history significant for hypertension and no prior history of abdominal surgery received treatment for a thoracolumbar epidural abscess including a partial corpectomy and reconstruction with titanium cage with autografting that involved harvesting a 5 cm long piece of bone from her right iliac crest through a posterior vertical incision (Figure 1). The patient returned to the medical center 19 months later with complaints of a three-day history of a spontaneous and increasingly painful baseball sized bulge in her right lumbar region. The patient denied any specific activity at the onset of pain. Physical examination revealed a reducible mass deep to the incision site scar from the previous bone harvest. Computed tomography revealed an incisional herniation of the right kidney immediately superior to the iliac crest at the location of bone harvest (Figures 2 and 3). The patient had a BMI of 18.0 and her blood pressure on initial presentation was 181/95, which was medically corrected prior to the repair. Laboratory studies did not demonstrate any change in kidney function.

An open lumbar incisional hernia repair was performed through the prior incision site scar. The renal mass was palpated through the herniation sack, the muscle edges were cleaned and exposed, and an 8.6 cm Parietex™ Composite Ventral Patch was placed subfascially and secured utilizing 8 interrupted 0 PDS® sutures. The subcutaneous tissue was closed using absorbable suture and the skin was closed utilizing Ethicon 4-0 Monocryl®. The patient progressed well postoperatively and demonstrated no hernia recurrence.

## 3. Discussion

Despite their popularity and perceived relative ease of access, iliac crest bone harvesting procedures have been reported to have an overall complication rate as high as 49% [3]. Incisional herniation of gastrointestinal structures following iliac crest bone harvesting was first reported in 1945 by Oldfield [4] and is reported to occur at frequent rates of 5%–9%, but their appearance in the literature is relatively rare with only a few hundred cases described [5]. Known risk factors for herniation of abdominal contents include obesity, increased intra-abdominal pressure, age greater than 65 years, hypertension, emergency surgery, gastrointestinal cancers, and mechanical ventilation [1]. However, many cases are not fully explained by these risk factors, as demonstrated by the patient in this article with low body weight, and the risk of herniation should not be prematurely discounted.

A variety of procedural solutions to reduce the occurrence of incisional herniation of gastrointestinal structures have been proposed, including bicortical bone grafts taken from the inner table and sparing the outer table [1], intracortical iliac crest bone grafting [6], and utilizing minimally invasive surgery when appropriate [7]. Despite these proposals to reduce complications, a broadly accepted approach has not been established to avoid iliac incisional herniation and damage to the abdominal contents, not considering the other serious potential complications in addition to herniation, including injury to various significant arteries and nerves, ureteral injury, infection, fracture, pelvic instability, hematoma, or chronic pain [1, 3].

While the iliac crest is an excellent source for autogenous bone, the current harvesting process has been shown to develop complications that are frequently underestimated in both frequency and severity as stated in the Introduction. Since the harvest is typically an adjunct procedure to the primary treatment for the patient, the high complication rate may result from reduced attention paid to the procedure, as demonstrated through low quantity of publications on the topic and through scarce detail in surgeon charting relative to the primary procedure. This lack of attention may stem from unapparent negative outcomes in the postoperative and short-term follow-up period [2], causing the procedure to be viewed as simple or benign relative to the primary operation.

While low body weight is not a common risk factor for herniation of abdominal contents, it is a risk factor for nephroptosis or “wandering kidney.” The condition is believed to be related to resorption of perirenal fat and is observed in up to 20% of thin females and on the right side 70% of the time [8]. Although the presented patient appeared underweight, preoperative imaging only focused on the area being treated in the spinal surgery. Imaging failed to allow certainty of the location of the kidney or adequate evaluation for potential concerns near the iliac crest. This lack of diagnostic evaluation relative to the effort placed on the primary procedure further justifies the concern that the adjunct procedure was viewed lightly.

Surgeons performing iliac crest bone harvest procedures are encouraged to provide thorough documentation on their preoperative evaluation, approach, process, and closure of the harvest. Future retrospective analyses can determine which factors from these procedures are related to disproportionate levels of negative outcomes. This could be comparable to known risk factors for patient demographics, which are more consistently reported [2]. With better surgeon procedure documentation and future research, either the common technique for iliac crest bone harvest procedures will be refined or the value of autografting will be reevaluated to establish if the benefit is truly worth the risks [9].
